# Communicable Diseases (Including COVID-19)—Induced Global Depression: Caused by Inadequate Healthcare Expenditures, Population Density, and Mass Panic

**DOI:** 10.3389/fpubh.2020.00398

**Published:** 2020-08-18

**Authors:** Muhammad Khalid Anser, Zahid Yousaf, Muhammad Azhar Khan, Abdullah Zafar Sheikh, Abdelmohsen A. Nassani, Muhammad Moinuddin Qazi Abro, Khalid Zaman

**Affiliations:** ^1^School of Public Administration, X'an University of Architecture and Technology, Xi'an, China; ^2^Higher Education Department Khyber Pakhtunkhwa, Government College of Management Sciences, Abbottabad, Pakistan; ^3^Department of Economics, University of Haripur, Haripur, Pakistan; ^4^Faculty of Business Administration, Institute of Business Administration, Karachi, Pakistan; ^5^Department of Management, College of Business Administration, King Saud University, Riyadh, Saudi Arabia

**Keywords:** communicable diseases, (COVID-19), healthcare expenditures, poverty incidence, population density, global depression, GMM estimator

## Abstract

Coronavirus (COVID-19) is spreading at an enormous rate and has caused deaths beyond expectations due to a variety of reasons. These include: (i) inadequate healthcare spending causing, for instance, a shortage of protective equipment, testing swabs, masks, surgical gloves, gowns, etc.; (ii) a high population density that causes close physical contact among community members who reside in compact places, hence they are more likely to be exposed to communicable diseases, including coronavirus; and (iii) mass panic due to the fear of experiencing the loss of loved ones, lockdown, and shortage of food. In a given scenario, the study focused on the following key variables: communicable diseases, healthcare expenditures, population density, poverty, economic growth, and COVID-19 dummy variable in a panel of 76 selected countries from 2010 through 2019. The results show that the impact of communicable diseases on economic growth is positive because the infected countries get a reap of economic benefits from other countries in the form of healthcare technologies, knowledge transfers, cash transfers, international loans, aid, etc., to get rid of the diseases. However, the case is different with COVID-19 as it has seized the whole world together in a much shorter period of time and no other countries are able to help others in terms of funding loans, healthcare facilities, or technology transfers. Thus, the impact of COVID-19 in the given study is negatively impacting countries' economic growth that converts into a global depression. The high incidence of poverty and social closeness increases more vulnerable conditions that spread coronavirus across countries. The momentous increase in healthcare expenditures put a burden on countries' national healthcare bills that stretch the depression phase-out of the boundary. The forecasting relationship suggested the negative impact of the coronavirus pandemic on the global economy would last the next 10 years. Unified global healthcare policies, physical distancing, smart lockdowns, and meeting food challenges are largely required to combat the coronavirus pandemic and escape from global depression.

## Introduction

**Figure 1:**
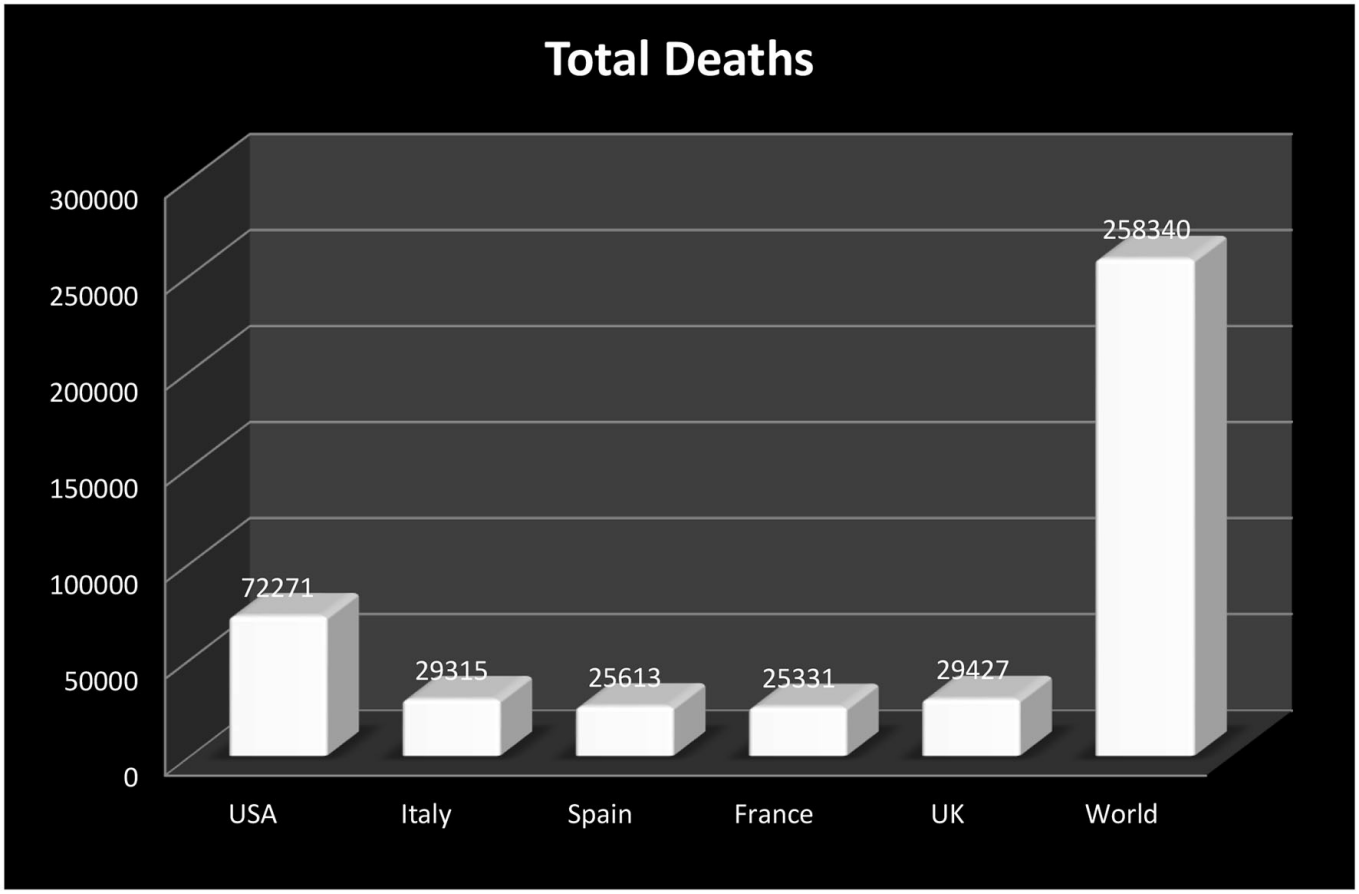
Death tolls by coronavirus in the five most affected countries. Source: Worldometer (May 06, 2020, 04:06 GMT).

Communicable diseases are not novel for the world; governments have learned from different infectious diseases in the past, such as Human Immunodeficiency Virus (HIV), Tuberculosis (TB), Ebola, and Spanish influenza a century before. The history of communicable diseases dates back much further, however we have only reported on the past 100 years. The 1918 influenza pandemic is considered to be one of the most deadly epidemics in recent history, which affected about one-third of the world's population, with a death toll of at least 50 million people globally. The list of communicable diseases is long, as more than 80 infectious diseases across the globe have been reported to date. The United States was largely affected by Hemagglutinin Type 1 and Neuraminidase Type 1 (H1N1) virus, where the death toll exceeded 670,000 people (1–3). Four decades later, the world was again hit by another communicable disease in 1957 with a new mutant influenza A, which is caused by an Hemagglutinin Type 2 and Neuraminidase Type 2 (H2N2) virus that spread from East Asia, also called “Asian Flu.” Asian influenza -A is different from HINI virus, as it is comprised of two different genes, i.e., hemagglutinin genes (H2), and neuraminidase (N2) genes. The virus was reported in Singapore first in February 1957, and hit the US in the summer of the same year; the world death toll from this virus was 1.1 million people, out of which the death toll around 116,000 in the US, making it the worst hit affected country [(4–6), etc.]. The pandemic did not end as its mutation, caused by influenza -A (1968 pandemic), caused by a Hemagglutinin Type 3 and Neuraminidase Type 2 (H3N2) virus. This virus was comprised of H3 hemagglutinin and contained the N2 neuraminidase from Asian influenza 1957. It was reported in the US in September 1968, and largely affected the older population; the median age was 65 years and above. The worldwide death toll exceeded 1 million and about 10% of the death toll was reported in the US alone. This virus continued to move worldwide as a seasonal flu that led to severe illness [(7–9), etc.]. This virus did not end, as in the spring of 2009, a new mutant influenza -A caused by H1N1 virus was detected in the US and quickly spread around the world. An estimated range of 151,700–575,400 people globally died from this pandemic virus infection in the first year. It is a seasonal virus that causes serious illness and increases hospitalizations and mortalities [(10, 11), etc.]. On May 12, 2009, A(H1N1)pdm09 pandemic was detected in two imported cases in Thailand, which increased up to 12 cases by the end of the month, and by July the virus had been transmitted and detected in all Thai provinces, which increased the death toll up to 65. The pandemic waves followed two irregular interval periods, which started from May 2009, maxing out in July and falling in December, while the second wave began in early January 2010, maxing out in February and ending in April. In between the 2 year time period, around 234,050 registered influenza cases were reported in the country, 47,433 of which were confirmed virus patients with A(H1N1)pdm09 infections, and the death toll reached 347 (12). The Ebola outbreak that was experienced in West Africa in March 2014, affected a number of affiliated bordering countries. More than 25,000 cases were registered and more than 10,000 deaths were reported with this virus. However, with unified healthcare policies and strengthening response capacities, the affected countries limited the transmission of the deadly disease in a given course of time [(13, 14), etc.]. Unified healthcare policies are desirable to improve countries' economic growth (15, 16). In late December 2019, Wuhan city in China detected a novel coronavirus (COVID-19) that threatened human lives; to date (25th April, 2020) COVID-19 has affected 2,831,915 people across 210 countries. The death toll exceeds 197,318 people, while the recovered cases are 807,037 across the globe (17). The WHO has declared an alert about this global pandemic, which represents a large family of viruses and causes serve respiratory problems like SARS, MERS, etc. The COVID-19 virus is a mutant strain of the coronavirus family, known as SARS-CoV-2. Figure 1 shows the total death tolls reported in the five most affected countries by COVID-19 for easy reference.

Since the emergence of coronavirus, a great amount of scholarly writing has been done on the given issue. For instance, Lai et al. (18) collected a cross-sectional data of 1,257 healthcare workers working in 34 different Chinese hospitals and analyzed their mental health states after handling coronavirus patients. The results suggested that, as healthcare workers are directly exposed to the coronavirus, there is a high need for physiological support and interventions to take care of frontline workers to reduce the symptoms of distress, insomnia, depression, anxiety, etc. Phelan et al. (19) argued that coronavirus was spreading all over the world from China, thus there was a need to handle this outbreak with global healthcare governance and strategies, including surveillance, testing, treatments, cooperation, technology transfers, and healthcare information. Wang et al. (20) discussed the early transmission channel of coronavirus in Wuhan city in China by considering a single center case study of 138 patients infected with 2019 Novel Coronavirus (2019-nCoV). The statistics show that the rate of patients administered to the Intensive Care Unit (ICU) were 26% of the total, while the death toll was 4.3%. The hospital associated human-to-human transmission rate was suspected to be 41%. The study concludes that the risk of transmission of coronavirus could not be analyzed as it was becoming increasingly dangerous as the weeks progressed. Livingston and Bucher (21) concluded that the coronavirus pandemic spread with an enormous rate despite aggressive control efforts. The study argued that the case-fatality ratio is higher in the elderly population, with a median age of more than and equal to 60 years. Italy is highly infected with coronavirus, which is an issue that needs to be taken seriously and controlled with effective interventions and surveillance. Torales et al. (22) reviewed the coronavirus associated studies and confirmed the psychological illnesses that were reported in the healthcare workers, suspected patients, and the general public. The results derived that the coronavirus outbreak is leading to additional health problems, including fear of death, anxiety, depression, insomnia, anger, etc. The need for efficient psychotherapy in suspected patients and counseling to the general masses would have a positive impact on reducing the risk of transmission of this disease. Table 1 shows the recent pieces of literature on the interlinkages between the coronavirus pandemic and economic activities all across the globe.

**Table 1:** Literature on coronavirus effect on economic activities across countries.

**References**	**Country**	**Economic sector**	**Results**
Gormsen and Koijen (23)	European Union and the US	Finance: stock prices	The forecast about dividends share dropping to 17 and 28% in the US and EU, respectively, is likely due to coronavirus. Economic growth is expected to further decrease growth by 3.8 and 6.3% in the US and the EU, respectively.
Hasanat et al. (24)	Malaysia	e-business	Online business is affected by the coronavirus pandemic due to lockdown, low sales and purchase, less buying intensions, supply chain issues, fear, etc.
Odhiambo et al. (25)	Kenya	Agriculture services, and the manufacturing sector	Due to the coronavirus outbreak, the agriculture sector decreased the share of 5.65% in total GDP, subsequently, tourism, construction, infrastructure development, and manufacturing dropped their share at around 1.35, 1.1, 2.06, and 0.85%, respectively.
Fernandes (26)	30 countries	Economic growth and services sector	It is predicted that in the mild scenario, economic growth will drop in the range of between 3 and 6%, depending upon the country's profile, while in the given sample of 30 countries, the median drop in GDP is expected to be −2.8% in 2020. The service sector is also affected due to breakdowns in the supply chain process, which tends to decrease economic growth in the crisis period as expected between 2.5 and 3% per month.
Huang et al. (27)	China	SMEs business	Due to the coronavirus pandemic, the SMEs sector has been badly affected and is highly dependent upon government support in terms of tax rebates, reduction in tax duties, provision of subsidies, flexible repayment of loan schedules, low interest rates, liquidity support, etc.
Fornaro and Wolf (28)	Worldwide	Economic growth	Macroeconomic policies would largely support country's economic growth during the crisis period associated with coronavirus
Bandyopadhyay (29)	Global evidences	General discussion on economy	The closure of educational institutions, travel restrictions, hospitality industry, financial, and related markets has caused economic declines across the globe.
Rodela et al. (30)	Developing countries	Healthcare sector	The coronavirus outbreak increases the high out-of-pocket healthcare expenditures that increases poverty incidences across countries.
Nseobot et al. (31)	Nigeria	Trade	Due to the coronavirus outbreak, a unit decrease in oil price put a stress on the economic growth by 0.005 units.
Isaifan (32)	China	Environment	The death toll from coronavirus has not exceeded 3.4% globally, whereas the death rate increase by air pollution was about 7.6% in 2016 worldwide. Due to lockdown, many polluting industries were temporarily shut down, which decreased N_2_O emissions and carbon emissions by 30 and 25%, respectively.

The study is important in the given circumstances, where coronavirus fear and depression have appeared around the world, creating chaos among community members as they seek remedial actions to get rid of the pandemic (33). A few policy actions have been derived by the international community to prevent the epidemic, including maintaining physical/social distancing among community members, increasing healthcare expenditures, and reducing poverty and hunger. This study has included all these factors and has examined their impact on the country's economic growth, which considers a proxy for economic suffering leads to a depression. The epidemic proportionally affected developed and developing countries, therefore, the current studies included both developed and developing countries in a panel of 76 selected countries during 2010–2019. The outbreak of coronavirus creates many healthcare issues, including inadequate healthcare equipment, patents' facilitation centers, quarantine issues, fear, depression, and many other sanitation issues that cause the situation to worsen. The global depression phase becomes lengthier if these critical issues are unresolved. This study intended to explore the answers to the following critical questions: *do communicable diseases, including COVID-19, exert a greater magnitude of stress in terms of negatively affecting countries economic growth which then converts into global depression?* The second question is *whether high population density and poverty incidence may increase the length of the coronavirus pandemic around the globe?* And finally, *how may we reduce human suffering and death tolls from the coronavirus plague across countries?* Knowing the answers to these questions will aid in helping the world with the coronavirus outbreak and stabilize the world from depression. In a given context, the study prepared a set of research objectives to analyze global depression through some policy instruments, including healthcare expenditures, population density, and poverty incidence in a panel of 76 countries. The research objectives are:

- To examine the impact of communicable diseases, including (COVID-19), on a country's economic growth.
- To investigate the role of healthcare expenditures in reducing the coronavirus outbreak
- To observe the changes in poverty rates and population density due to the coronavirus pandemic on economic growth across countries
- To determine the inter-temporal relationship between the coronavirus pandemic and economic growth over a time horizon.

These objectives have been set and analyzed by using sophisticated econometric techniques in order to reach some conclusive findings.

## Data Source and Methodology

The study used the following key factors that affect a country's economic growth and which turn into economic losses during the outbreak of communicable diseases, including COVID-19. Economic growth (denoted by EG) is used as a proxy variable for analyzing economic losses due to an emerging epidemic, which served as a response variable. The data of GDP per capita in constant 2000 US$ is used in the given analysis. The explanatory variables are as follows: poverty incidence (denoted by PI) is used to get an insight into the “mass panic” among the country's residents during the coronavirus pandemic, as poor populations are directly exposed to communicable diseases caused by a lack of knowledge, low/no direct income, persistent unemployment, and inadequate healthcare facilities. This restlessness then creates more panic during the emergence of the epidemic that negatively affects the country's economic growth. The headcount ratio in percentage form is used for this reason. COVID-19 (denoted by COVID-DUM) is used to assess the magnitude and the intensity of coronavirus that largely increases due to high social contact between the population members, as this virus easily spreads through close contact in the community, like, handshaking, sneezing, coughing, touching, etc., hence it is highly possible to get infected with the virus when people per square km of the land area are living in compact places. Thus, the COVID-DUM is formed and assigned values of 1 and 0. The COVID-DUM value 1 represents the likely occurrence of coronavirus when the population density is in triple digits (i.e., 100 people per square km of land area) and 0 represents otherwise. The COVID-DUM data is extracted from the data set of population density (denoted by PD), which is further included in the regression estimates to get more insight into social distancing. The data of deaths caused by communicable diseases (denoted by CD) as a percentage of total deaths and per capita healthcare expenditures (denoted by HE) as in US$ is added to the study to minimize the probability of omission bias problems in the given model. Further, both the variables have important policy implications on the country's economic growth that can be used to assess global depression caused by insufficient healthcare expenditures, which links to the increasing cause of deaths by communicable diseases including (COVID-19). Table A in the appendix shows the list of sample countries used in the study, which covered a period of 2010–2019. The data is taken from World Bank (34) and POVCAL Net database.

The strong viability of regressors and regressand in the given context need an empirical model that would facilitate answering the causes of global depression associated with high communicable diseases including COVID-19. The study utilized a traditional Solow growth model that considers a starting point for any growth—specific modeling, i.e.,

(1) $$\begin{array}{l} Y_{it}=\beta_{0}+\beta_{1}L_{it}+\beta_{2}K_{it}+\beta_{3}{\left(L\times T\right)}_{it}+\beta_{4}{\left(K\times T\right)}_{it}+\varepsilon_{it} \end{array}$$

Where Y shows economic output, L shows labor stock, K shows capital investment, T shows technology, i and t show cross-sections and time period, and ε shows error term.

Equation (1) shows the conventional style Solow growth model that comprises labor, capital, technology, and their resulting impacts on economic output. Further, the moderation effect of technology with labor and capital stock shows the labor—augmented technology and capital—augmented technology that would increase many times to the output through a multiplier effect. Equation (1) is modified and extended by the given set of parameters in order to get fresh insight into the real-time issue faced by the world regarding the coronavirus pandemic, i.e.,

(2) $$\begin{array}{l} \;EG_{it}=\beta_{0}+\beta_{1}COMD_{it}+\beta_{2}HE_{it}+\beta_{3}PD_{it}+\beta_{4}PI_{it}\\ \;+\beta_{5}COVID-DUM+\varepsilon_{it}\\ ∴{\left(\frac{\partial EG}{\partial COMD}\right)}_{it}<0,{\left(\frac{\partial EG}{\partial HE}\right)}_{it}<0,{\left(\frac{\partial EG}{\partial PD}\right)}_{it}<0,{\left(\frac{\partial EG}{\partial PI}\right)}_{it}\\ \;<0,{\left(\frac{\partial EG}{\partial COVID-DUM}\right)}_{it}<0 \end{array}$$

Where EG shows economic growth, COMD shows communicable diseases, HE shows healthcare expenditures, PD shows population density, PI shows poverty incidence, COVID-DUM shows COVID dummy, i and t show 76 countries and time period from 2020 to 2019.

**Figure 2:**
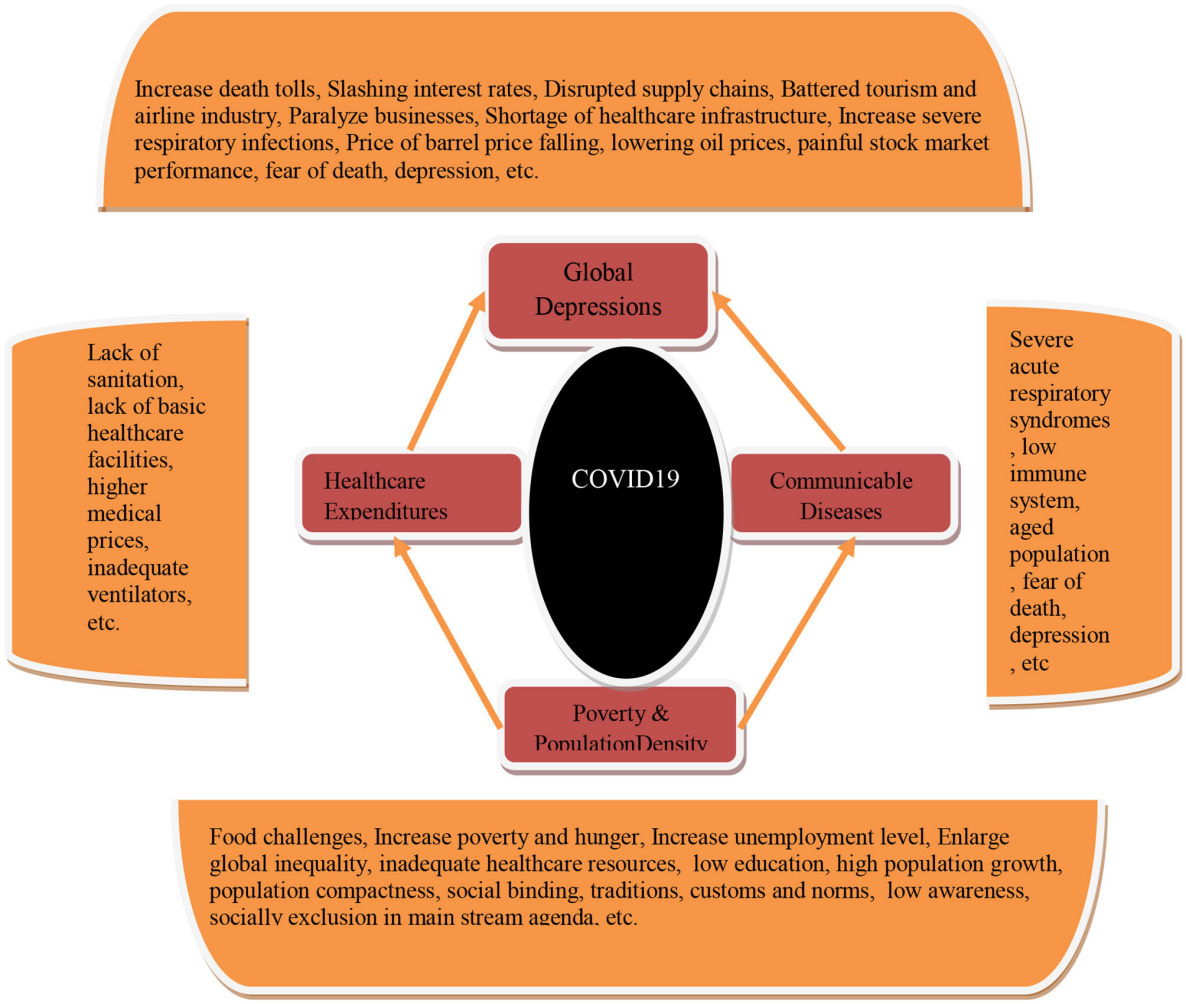
Research framework. Source: Self extract.

Equation (2) shows that it is likely that communicable diseases, including COVID-19, will increase economic suffering in the form of decreasing a country's economic output that will have a negative impact on the globalized world, which causes global depression. The other factors, including healthcare expenditures, population density, and poverty incidence, would likely place more pressure on economic output because of insufficient healthcare resources, highly-dense populations, and poverty and hunger. These factors are crucial and need a fair assessment in order to devise strong policies to reduce economic suffering caused by the coronavirus and other factors to lessen global depression through economic opportunities. Figure 2 shows the research framework of the study.

Figure 2 shows the different causes of global depression that are interconnected with poverty incidence, communicable diseases, population density, and healthcare resources. The blend of efficient healthcare, economic, social, and environmental policies are largely desirable to escape from this pandemic with the adoption of curative and preventative policies across the globe.

The stated objectives need to be checked by sophisticated econometric techniques to get fresh evidence about global depression due to the outbreak of coronavirus. The study employed a differenced panel GMM estimator. This technique is utilized on longitudinal data sets where the cross-section identifiers are greater than the time period that is used in this study, i.e., Cross-sections consist of 76 selected countries while data is used from 2010 to 2019. The second reason is that the differenced GMM estimator controls for possible endogeneity issues and serial correlation issues from the model. Third, it includes the dynamic nature of the regressand in the list of regressors, where the regressand is included with the regressors to analyze the initial convergence in the growth model. Fourth, the list of regressors can be further utilized as instrumental variables added by their first lagged, hence it can control for possible endogenous issues and autocorrelation issues in the model, and finally, the validity of using regressors as an instrumental variable by their first lagged is a real challenge to check whether the given instruments are reliable or not. For this purpose, the J-statistic and instrumental ranks are used to determine its validity. These features give clear distinctions from the rest of the instrumented techniques, for instance, simple OLS, two-stage least squares, three-stage least squares, and simple GMM estimates with fixed and random effect. Further, the study benefits from using the innovation accounting matrix that consists of two basic inter-temporal techniques, i.e., impulse response function (IRF) and variance decomposition analysis (VDA). The technique is based upon both the VAR specifications and determined by the shocks pertaining to the regressand by their set of regressors over a time horizon. Thus, it specified the nature and magnitude of the explanatory factors to the outcome variable in forecasting apparatus.

## Results and Discussion

Table 2 shows the descriptive statistics of the candidate variables and found that a country's economic growth and healthcare expenditures have a mean value of US$17,230.83 and US$1,474 with a maximum value of US$110,742 and US$9,871.74, and a minimum value of US$341.55 and US$15.12, respectively. The statistics clearly show that the panel consists of all the representatives of the countries across the globe where high-income to low-income countries have been included in the given model to give equal rank to all of them without any special attention. This uniqueness gives reliable estimates and provides evidence for both sides of the coin. The maximum count of deaths caused by communicable diseases is about 71.500% of total deaths, with a mean value of 13.078 %. Poverty incidence shows the maximum value of 72.3% with a mean value of 15.395%. The data of COVID-19 dummy is extracted from the population density data, as population density shows that the selected panel of countries has a high to low dense population data; the highest value is 2017.274 people per square km of land area while the lowest value is 1.750. Thus, on the basis of ranking the population density data, the COVID-DUM is assigned a value 1 to those countries where the population density is more than and equal to three digits, i.e., 100, while 0 is assigned otherwise. The mean value of COVID-DUM shows 0.349, which depicts that on average 35% of the countries in the selected panel have a population density that is more than or equal to three digits, while 65% of countries have a population compactness that is limited to two digits.

**Table 2:** Descriptive statistics.

**Methods**	**EG**	**CD**	**HE**	**PD**	**PI**	**COVID-DUM**
Mean	17230.83	13.078	1474.137	145.428	15.395	0.349
Maximum	110742.3	71.500	9871.742	2017.274	72.300	1
Minimum	341.554	1.200	15.126	1.750	0.010	0
Std. Dev.	19392.71	15.423	1936.618	266.143	14.956	0.477
Skewness	1.929	2.181	1.790	4.499	1.192	0.631
Kurtosis	7.594	7.058	5.755	25.494	4.636	1.399

*EG, economic growth; CD, communicable diseases; HE, healthcare expenditures; PD, population density; PI, poverty incidence; COVID-DUM, dummy variable*.

Table 3 shows the differenced panel GMM estimates and found that communicable diseases other than coronavirus increase the country's economic growth in the form of receiving aid and other technology transfers from the rest of the world. This aid has controlled or reduced the intensity of some infectious diseases, like Ebola, hepatitis, flu, tuberculosis, measles, rabies, Zika, etc. However, the novel coronavirus has largely affected the whole world and the world's biggest economies, including the United States and other European countries that [(35–37), etc.]. The new mutant coronavirus evenly affected rich and poor nations without any discrimination. The role of technology transfer, healthcare facilities, and other socio-economic reforms have been taken under consideration and the whole world should make unified policies to control this epidemic and get out from global depression [(38, 39), etc.].

**Table 3:** Differenced panel GMM estimates.

**Variables**	**Coefficient**	**Std. error**	**t-statistic**	**Prob**.
**Dependent variable: EG**				
LNEG(-1)	0.814	0.006	129.142	0.000
LNCD	284.784	87.635	3.249	0.001
LNHE	−1.633	0.083	−19.495	0.000
LNPD	6.404	3.160	2.026	0.043
LNPI	−180.408	72.790	−2.478	0.013
COVID-DUM	−2348.692	1303.121	−1.802	0.072
**Diagnostic tests**				
J-statistic	26.622	Instrument rank		33
Prob(J-statistic)	0.484295			
**Arellano-bond serial correlation test**				
AR(1)	m-statistic (Prob. Value)	−1.220 (0.221)		
AR(2)		−2.447 (0.014)		

*EG, economic growth; CD, communicable diseases; HE, healthcare expenditures; PD, population density; PI, poverty incidence; COVID-DUM, dummy variable*.

The results of this study further show that healthcare expenditures enormously increase in a given scenario that increases the rate of global depression. Most of the countries have been unable to access basic healthcare facilities, including less availability of soap and water for handwashing, less access to ventilators, surgical masks, test swabs, gowns, and other protective surgical equipment [(40–43), etc.]. Besides healthcare expenditures, there are many other factors that affect coronavirus, including increased social contact between community members due to population compactness, although it increases economic growth in terms of saving land resources and devoting it to cultivation and forestry, while on the other side, it becomes a major issue to prevent the outbreak of coronavirus on a global scale. Governments have taken many measures for controlling the coronavirus, including creating social awareness, social distancing, lockdowns, online work, social and print media campaigns, charity shows, fundraising, and many more options, however, due to spreading mass panic among the community members, this has negatively affected the country's economic activities [(44–47), etc.]. Poor nations are largely affected by the coronavirus pandemic, as they already have fewer resources, shortages of food, illiteracy, low health profile, unemployment, lack of a voice, inadequate social safety nets, and many other vulnerabilities that negatively affect the country's economic growth and exacerbate coronavirus [(48–52), etc.].

After obtaining the parameter estimates of the studied coefficients, there is a greater need for exploring the forecasted (inter-temporal) relationship between the stated variables for the next 10 years. For this purpose, the study used innovation accounting matrix, which is based upon two innovative functions, impulse Response Function (IRF) and Variance Decomposition Analysis (VDA). The IRF estimates assist to identify the direction of the stated variables that could be seen by various economic and healthcare shocks over a time horizon. On the other side, the VDA estimates showed the magnitude of the candidate variables on the response variable over time. Thus, both innovation estimates help to determine future preventive strategies to minimize the COVID-19 pandemic across countries. Table 4 shows the IRF estimates for easy reference.

**Table 4:** Estimates of impulse response function.

**Response of EG**						
**Period**	**EG**	**CD**	**HE**	**PD**	**PI**	**COVID-DUM**
2020	661.423	0	0	0	0	0
2021	935.7011	7.859675	15.64205	−25.09291	−1.648502	4.965830
2022	1055.136	11.43912	24.25080	−61.87502	−1.358281	−1.237942
2023	1112.271	12.76133	30.45476	−105.5018	−0.216464	−10.17955
2024	1144.510	13.23417	35.68470	−154.2314	1.249298	−19.54193
2025	1166.959	13.39622	40.51965	−207.6275	2.782013	−28.38294
2026	1185.734	13.47229	45.18696	−265.8105	4.259219	−36.26393
2027	1203.323	13.56048	49.77130	−329.1579	5.615768	−42.94590
2028	1220.758	13.70810	54.29894	−398.1864	6.808375	−48.26264
2029	1238.479	13.94265	58.77175	−473.5089	7.800870	−52.06900

*EG, economic growth; CD, communicable diseases; HE, healthcare expenditures; PD, population density; PI, poverty incidence; COVID-DUM, dummy variable*.

**Figure 3:**
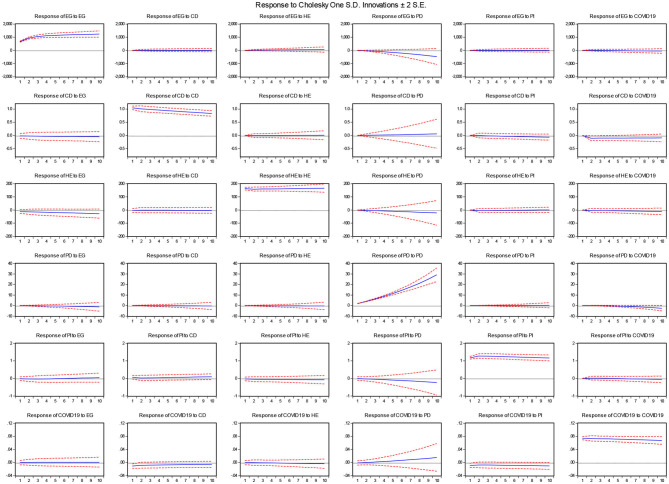
IRF estimates. Source: Author's estimation. EG, economic growth; CD, communicable diseases; HE, healthcare expenditures; PD, population density; PI, poverty incidence; COVID-DUM, dummy variable.

The estimates show that communicable diseases and healthcare expenditures will likely increase countries' economic growth over a time horizon, whereas population density and COVID-19 will mainly increase economic suffering in the form of decreasing economic output for the next 10 years. The poor income group experience decreased economic growth up to 2023 however, after 2023 it countries' economic growth begins to increase due to increased income inequality across countries. The rest of the effects can be seen in Figure 3 for easy reference.

**Figure 4:**
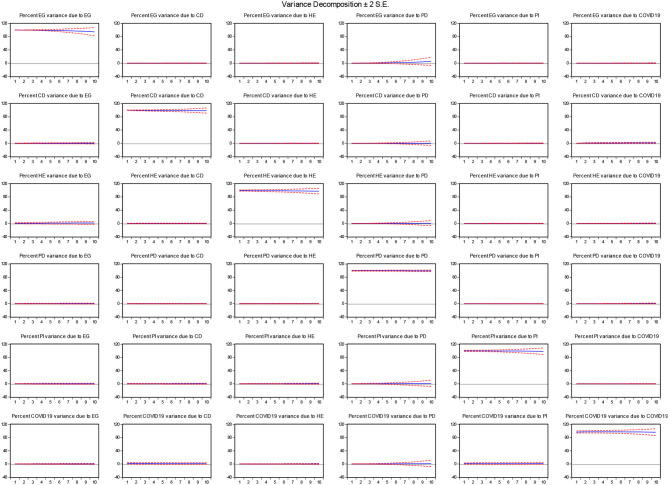
VDA estimates. Source: Author's estimation. EG, economic growth; CD, communicable diseases; HE, healthcare expenditures; PD, population density; PI, poverty incidence; COVID-DUM, dummy variable.

Table 5 shows the VDA estimates and suggests that population density will exert a greater magnitude on countries' economic growth with a standard error shock of 5%, followed by healthcare expenditures, COVID-19, and communicable diseases, while poverty incidence will have the least effect on countries' economic growth over a time horizon. The complete description can be visualized in Figure 4.

**Table 5:** Estimates of variance decomposition analysis.

**Variance Decomposition of EG**							
**Period**	**S.E**.	**EG**	**CD**	**HE**	**PD**	**PI**	**COVID19**
1	661.4232	100	0	0	0	0	0
2	1146.290	99.92667	0.004701	0.018621	0.047920	0.000207	0.001877
3	1559.437	99.77324	0.007921	0.034245	0.183325	0.000188	0.001077
4	1918.675	99.51542	0.009656	0.047816	0.423457	0.000125	0.003526
5	2239.830	99.13375	0.010577	0.060470	0.784880	0.000123	0.010200
6	2534.635	98.61151	0.011053	0.072778	1.283943	0.000217	0.020505
7	2811.503	97.93266	0.011279	0.084981	1.937372	0.000405	0.033302
8	3076.592	97.08100	0.011362	0.097138	2.762534	0.000672	0.047295
9	3334.626	96.03983	0.011362	0.109202	3.777410	0.000989	0.061207
10	3589.456	94.79214	0.011314	0.121056	5.000302	0.001326	0.073867

## Conclusions and Policy Implications

The world has been relentlessly affected by the outbreak of this deadly coronavirus, even though it is still only developing. In this study, a number of important factors have been identified, which might help researchers and policy makers to understand the emerging global depression. This study has selected a panel of 76 developed and developing countries in order to examine the vulnerabilities caused by coronavirus across all the segments of society. The overall results come to the following policy conclusions:

- Communicable diseases, including COVID-19, largely increase economic suffering through the increased demand for healthcare infrastructure, exacerbated by poverty incidence and social compactness. Thus, the need for healthcare technology transfers from developed to developing countries, fund allocation for poor nations to reduce global inequality which would help them out from poverty, hunger, and diseases, and smart cities planning would likely decrease the coronavirus pandemic across the globe.
- Global depression can be reduced by making unified healthcare policies, smart lockdowns, adopting easy fiscal and monetary policy instruments, and helping other nations through debt resettlement, resolving conflicts, and political stability to wrap up the pandemic.
- The provision of personal protective equipment, coronavirus screening laboratories, swab tests, ventilators, and quarantine facilities support the frontline doctors and paramedical staff in handling this epidemic and reducing the death toll.
- Domestic fundraising campaigns, charities, social awareness, guidelines of first-hand prevention, and social distancing would be appropriate measures to control the coronavirus pandemic.
- Special attention is required to protect local businesses by providing temporary relief to local businesses in the form of healthcare subsidies, tax rebates, and financial assistance. To lower the death toll it is necessary to spend money on coronavirus testing, screening, treating, and containing the epidemic. These actions may reduce the intensity and fear of the coronavirus pandemic, which would be helpful to control the outbreak of the disease.
- The advancement of new healthcare technologies are deemed desirable to diagnose and treat coronavirus.
- The training of doctors and paramedical staff should be the priority to handle coronavirus patients and discuss with them about symptomatic treatment and immunity boosters to get an increased chance of early recovery from this infectious disease.
- Population mixing is the main transmission route of spreading coronavirus from one person to another, thus there is a high need to raise awareness among community members to avoid massive gatherings. The government should have to take some initiatives for providing home-based jobs and given them enough salary to convince the massive population to stay at their homes.
- Extreme physical distancing options, including school closures, business closures, and travel restrictions, may result in a few early achievements as they raise awareness in the community about how to avoid this infectious disease, however, if these strategies are delayed, these activities should be substituted by other options, like online teaching, work at home, meetings conducted online, risk management, online training programs, and other social programs that a person can be engaged with and learn new things to resettle his/her self quickly in a new mode.
- The lower-income strata group will largely suffer from this pandemic due to low awareness, inadequate healthcare resources, unemployment, illiteracy, the absence of social safety net programs, a lack of voice, etc., and all these vulnerabilities will largely victimize poor people more than non-poor. Thus, there is a greater need to support poorer countries through cash transfer programs. The provision of basic food material, basic healthcare services, sanitation facilities, and proper counseling and guidance would minimize the risk of spreading infectious disease.

It is a reality in a given context that social/physical distancing and smart lockdowns exert a positive health effect, but these measures have potentially caused more economic suffering that will lead to a global depression. The disruption of the supply chain, fear of business losses, supply-demand production gap, and the global healthcare crisis will make this episode more painful. The need for joint global efforts, unified economic and healthcare policies, and subsidized economic sectors may decrease the intensity of the global depression and progress toward the eradication of the coronavirus. The basic limitation of the current study is inadequate data availability for COVID-19; hence, the study selected given countries on the basis of the country's economic growth per capita. The impact of COVID-19 on case per million and death per million is also important, which can be further explored in future studies.

## Data Availability Statement

Data is freely available at the World Development Indicators, published by the World Bank. https://databank.worldbank.org/source/world-development-indicators.

## Author Contributions

MKA: conceptualization, methodology, and supervision. ZY: software and formal analysis. MK: resources. AN: formal analysis, writing-reviewing, and editing. MMQA: visualization. KZ: data curation and validation. All authors: contributed to the article and approved the submitted version.

## Conflict of Interest

The authors declare that the research was conducted in the absence of any commercial or financial relationships that could be construed as a potential conflict of interest.

## Appendix

**Table A:** List of countries.

“Albania, Algeria, Armenia, Australia, Austria, Bahrain, Belarus, Belgium, Bolivia, Bosnia and Herzegovina, Brazil, Bulgaria, Canada, Chile, China, Colombia, Costa Rica, Croatia, Ecuador, Egypt, Estonia, Ethiopia, Finland. France, Georgia, Greece, Hungary, Iceland, India, Indonesia, Iran, Ireland, Italy, Japan, Jordan, Kazakhstan, Kenya, Korea, Kyrgyz Republic, Latvia, Lithuania, Luxembourg, Malaysia, Malta, Mauritius, Mexico, Mongolia, Morocco, Namibia, New Zealand, Panama, Peru, Philippine, Poland, Portugal, Romania, Russia, Senegal, Serbia, Slovak Republic, Slovenia, South Africa, Spain, Sri Lanka, Sweden, Tanzania, Thailand, Tunisia, Turkey, Ukraine, UK, USA, Uruguay, Vietnam, Yemen, and Zimbabwe”.
